# Development and Validation of a Deep Learning Radiomics Model Predicting Lymph Node Status in Operable Cervical Cancer

**DOI:** 10.3389/fonc.2020.00464

**Published:** 2020-04-15

**Authors:** Taotao Dong, Chun Yang, Baoxia Cui, Ting Zhang, Xiubin Sun, Kun Song, Linlin Wang, Beihua Kong, Xingsheng Yang

**Affiliations:** ^1^Department of Obstetrics and Gynecology, Qilu Hospital of Shandong University, Jinan, China; ^2^Cheeloo College of Medicine, Shandong University, Jinan, China; ^3^Department of Obstetrics and Gynecology, The Fourth People's Hospital of Jinan, Jinan, China; ^4^Pharmacy Department, Infectious Diseases Hospital of Jinan, Jinan, China; ^5^Department of Statistics, School of Public Health, Cheeloo College of Medicine, Shandong University, Jinan, China; ^6^Department of Radiation Oncology, Shandong Academy of Medical Science, Shandong Cancer Hospital Affiliated to Shandong University, Jinan, China

**Keywords:** cervical cancer, deep learning, radiomics, lymph node status, deep neural network

## Abstract

**Aim:** To develop and validate a deep learning radiomics model, which could predict the lymph node metastases preoperatively in cervical cancer patients.

**Patients and methods:** We included a cohort of 226 pathological proven operable cervical cancer patients in two academic medical institutions from December 2014 to November 2017. Then this dataset was split into training set (*n* = 176) and independent testing set (*n* = 50) randomly. Five radiomic features were selected and a radiomic signature was established. We then combined these five radiomic features with the preoperative tumor histology and grade of these patients together. Baseline logistic regression model (LRM) and support vector machine model (SVM) were established for the comparison. We then explored the performance of a deep neural network (DNN), which is a popular deep learning model nowadays. Finally, performance of this DNN was validated in another independent test set including 50 cases of operable cervical cancer patients.

**Results:** One thousand forty-five radiomic features were extracted for each patient. Twenty-eight features were found to be significantly correlated with the lymph node status in these patients (*P* < 0.05). Five radiomic features were further selected for further study due to their higher predictive powers. Baseline LRM incorporating these five radiomic and two clinicopathological features was established, which had an area under receiver operating characteristic curve (ROC) of 0.7372 and an accuracy of 89.20%. The established DNN model had four neural layers, in which layer there were 10 neurons. Adagrad optimizer and 1,500 iterations were used in training. The trained DNN had an area under curve (AUC) of 0.99 and an accuracy of 97.16% in the internal validation. To exclude the overfitting, independent external validation was also performed. AUC and accuracy in test set could still retain 0.90 and 92.00% respectively.

**Conclusion:** This study used deep learning method to provide a comprehensive predictive model using preoperative CT images, tumor histology, and grade in cervical cancer patients. This model showed an acceptable accuracy in the prediction of lymph node status in cervical cancer. Our model may help identifying those patients who could benefit a lot from radiation therapy rather than primary hysterectomy surgery if this model could resist strict testing of future randomized controlled trials (RCTs).

## Introduction

Cervical cancer has been ranked as the fourth most common cancer in women worldwide, where 85% of these patients occur in developing countries (1, 2). In these developing countries, cervical cancer is the leading cause of cancer related deaths (3). It is important to clarify the lymph node status preoperatively in cervical cancer, which could facilitate the treatment planning and prognosis making. Many pathological factors have been found to be correlated with lymph node metastasis in cervical cancer including the abnormal expression of TIMELESS, high expressed programmed death ligand 1 (PD1), and LNMICC expression elevation (4–6). However, these pathological factors could only be obtained postoperatively. On the other hand, preoperative imaging tests including CT scan and MRI exams may have some values in the prediction of lymph node metastases (7, 8). Still, previous study also showed that these radiological exams could not precisely identify the positive lymph node sufficiently in cervical cancer patients (9).

Radiomics have drawn much more attentions in recent years and it is a process of converting medical images into mineable high dimensional data in a high throughput manner which could be further analyzed for clinical decision making (10). The progress in pattern recognition and increased sample data in medical radiology has promoted the development of radiomics remarkably. Previous studies have shown that these objective and quantitative radiomic features could be used as meaningful biomarkers for the prediction of treatment response and prognosis in various types of cancer including the cervical cancer (11–15). However, the main limitation of popular radiomic studies is that the performance of these predictive model almost always could not reach the clinical useful level since they are often based on the conventional machine learning method and limited numbers of variables (16).

Deep learning (DL) is a subspecialty of the machine learning and artificial intelligence, which has shown impressive performance in medical diagnosis (17–19), treatment response prediction (20), and prognosis making (21–23). This new technique has two main advantages over the conventional machine learning: one is that DL could perform much better when it is used for larger samples, and second is that DL could incorporated more different types of data since it could automatically modulate its weights in the process of training (23). Yet, it is unclear how we could construct the optimal DL model in the radiomic studies, especially for the lymph node metastases prediction in cervical cancer.

Thus, we here explored the possibility of developing a deep learning radiomic model which could incorporate preoperative radiomic and clinicopathological features. The established model may be useful in identify those patients who would be spared for the unnecessary hysterectomy surgery in future.

## Materials and Methods

### Characteristics of Patients

This study retrospectively included 226 cases of operable cervical cancer patients from Qilu Hospital of Shandong University and Shandong Cancer Hospital between December 2014 and November 2017. These patients were divided into train set (*n* = 176) and test set (*n* = 50). In our study, we used the train set for the development of every predictive model, and the test set for the final independent validation of the deep neural network (DNN) model. This institutional review board of these two hospitals had approved this retrospective study. All subjects gave written informed consent in accordance with the Declaration of Helsinki. Inclusion criteria include the following: patients were aged 18 years or older, were diagnosed with stage IA to stage IIB cervical cancer confirmed by histopathology according to the FIGO staging system 2019, and received surgical resection without prior therapy. Exclusion criteria are patients with (1) autoimmune disease; (2) active infectious such as gastroenteritis, appendicitis, and cholecystitis.

### Clinicopathological Variables

For each patient, we collected the clinicopathological characteristics including age at diagnosis, smoke status, comorbidity, stage (24), tumor location, tumor diameter, depth of invasion, tumor histology, tumor grade, parametrial invasion status, margin involvement, resected lymph node numbers, Karnofsky performance scores (KPS). The outcome variable was lymph node metastasis status, which was also collected for each patient.

### Image Segmentation and Radiomic Feature Extraction

**Figure 1:**
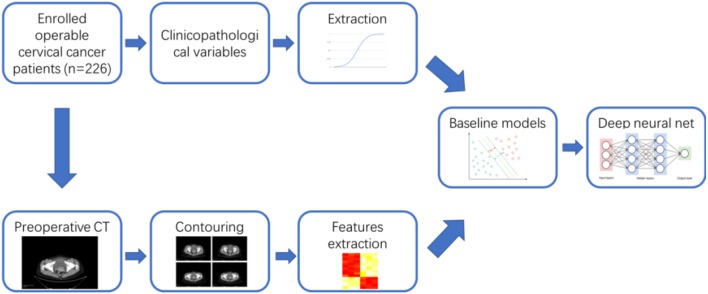
Global analysis pipeline.

For each patient, we have collected the corresponding preoperative contrast-enhanced CT images for abdomen and pelvis. The overall workflow of this study was illustrated in Figure 1. For each patient, one image containing typical lesion was chosen for further study. 3D gross tumor volume (GTV) was manually segmented and delineated by two expert radiologists independently using the 3D Slicer software (version 4.10, http://www.slicer.org).

In total, 1,045 CT image radiomic features including intensity, shape, texture, wavelet, and log transformation were extracted for each of the 226 cervical cancer patients. The public radiomic study platform was used for the radiomic feature extraction (https://www.radiomic.io). One thousand forty-five radiomic features were extracted for each patient based on the open source Pyradiomics platform (www.radiomics.io), in which those features could be divided into five categories including the intensity, shape, texture, wavelet, and log transformation. The detailed description of these radiomics features could also be found in our previous work (15).

### Training and Validation of the Deep Learning Model

The selected radiomic features and clinicopathological variables were used as the input of a deep neural network which was constructed by the DNNClassifier Custom Estimator from the TensorFlow open-source framework (v1.3, Google, Mountain View, California, USA) (25). Training and testing of the DNN were performed on a Linux Ubuntu 17.04 workstation. This station has a 2.6 GHz Core i7-9750H CPU and a Nvidia N18E-GO Graphics.

Various combination of hyperparameters were explored including the batch size, layers of the network, neurons in each layer, etc. We did not explore very deep neural network (>10,000 layers) based on the consideration of computation cost. The best network architecture was selected after numerous explorations. The final DNN was a balance between computation cost and model performance, which had four hidden layers and 10 neurons in each layer. The Rectified Linear Unit (ReLU) function was used for activation because this activation function could improve the computing speed. Adagrad optimizer was used for the gradient descent since it was suitable for the sparse data. One thousand five hundred iteration was used and increased iteration times could no longer improve the model performance. The output of the DNN was binary data, in which zero was assigned to negative lymph node metastasis and one was assigned to positive lymph node metastasis.

### Statistical Analyses

#### Important Radiomic Features Selection

Stable radiomic features were selected from the results of two delineation using the intraclass correlation analyses, and only 118 features that have more than 0.8 intra-class correlation (ICC) coefficient were selected. Logistic regression analyses were then used for selecting the predictive radiomic features. Twenty-eight features had *p* < 0.05, among which five features were selected for further analyses due to their higher C index values (15). Radiomic signature was then established using the multivariate logistic regression model.

#### Baseline Model Establishment

Multimodal features including radiomic, and clinicopathological variables were incorporated into a single model based on the multivariate logistic regression model 5-fold cross validation was also used for these analyses. Performance of this model was evaluated with the accuracy, sensitivity, specificity, and AUC values.

All statistical analyses are two-sided, with the significance level of 0.05. Statistical analyses were performed with “rms,” “Hmisc,” and other modules in R programming language and environment (http://www.r-project.org). “svm,” “confusion_matrix,” “roc_curve,” and “auc” modules in Sci-Kit Learn (www.scikit-learn.org) were used in the SVM establishment and other analyses. The STATA software (version 14.1) was also used in the statistics.

## Results

### Characteristics of the Patients

Table 1 has shown the baseline characteristics of cervical cancer patients in train set (*n* = 176) and independent test set (*n* = 50). We consecutively included 226 available cervical cancer patients for the development and validation of the model. The total dataset was split into training set and validation set randomly using the “Train_test_split” model in the sklearn framework. In the train set, 18 cases were staged as IA (10.2%), 74 cases were 1B1 (42.0%), 30 cases were 1B2 (17.0%), 45 cases were 2A (25.6%), and 9 cases were 2B (5.1%). One hundred forty-four cases were squamous cell cancers (81.8%), 24 cases were adenocarcinoma types (13.6%), and eight cases were other histological types (4.5%). Fifty-five cases (31.3%) had the high grade in histology, 55 cases (31.3%) had the moderate grade, and 66 cases (37.5%) had the low grade. Twenty-six cases (14.8%) had the lymph node metastases in the train set. The test set included 50 cases of operable cervical cancer patients. The clinicopathological variables between train set and test set have no statistically significant difference (*P* > 0.05). Figure 1 had shown the overall analyses pipeline of this study.

**Table 1:** Patients characteristics.

**Characteristic**	**Train set (*****n*** **=** **176)**		**Test set (*****n*** **=** **50)**		
	**No**.	**%**	**No**.	**%**	***P*-value**
**Age, years**					
Mean	45.6		47.2		>0.05
SD	9.9		8.3	
**Smoke**					
No	171	97.2	47	94.0	>0.05
Yes	5	2.3	3	6.0	
**Comorbidity**					
No	118	67.0	35	70.0	>0.05
Yes	58	33.%	15	30.0	
**Morbidity**					
No	124	70.5	36	72.0	>0.05
Yes	52	29.5	14	28.0	
**Stage**					
1A	18	10.2	6	12.0	>0.05
1B1	74	42.0	23	46.0	
1B2	30	17.0	8	16.0	
2A	45	25.6	12	24.0	
2B	9	5.1	1	2.0	
**Positive pelvic lymph node**					
No	150	85.2	42	84.0	>0.05
Yes	26	14.8	8	16.0	
**Resected lymph node numbers**					
Mean	19.6		21.2		>0.05
SD	7.0		7.4		
**Tumor diameter, cm**					
Mean	3.1		3.2		>0.05
SD	1.5		2.6		
**Depth of invasion**					
Inner 1/3	48	27.3	15	30.0	>0.05
Middle 1.3	44	25.0	13	26.0	
Outer 1.3	84	47.7	22	44.0	
**Histology**					
Squamous	144	81.8	43	86.0	>0.05
Adenocarcinoma	24	13.6	5	10.0	
Others	8	4.5	2	4.0	
**Differentiation**					
Poor	55	31.3	17	34.0	>0.05
Moderate	55	31.3	16	32.0	
Well	66	37.5	17	34.0	
**Parametrial involvement**					
No	168	95.5	48	96.0	>0.05
Yes	8	4.5	2	4.0	
**Margin involvement**					
No	171	97.2	49	98.0	>0.05
Yes	5	2.8	1	2.0	

### Radiomic Features Selection and Signature Establishment

**Figure 2:**
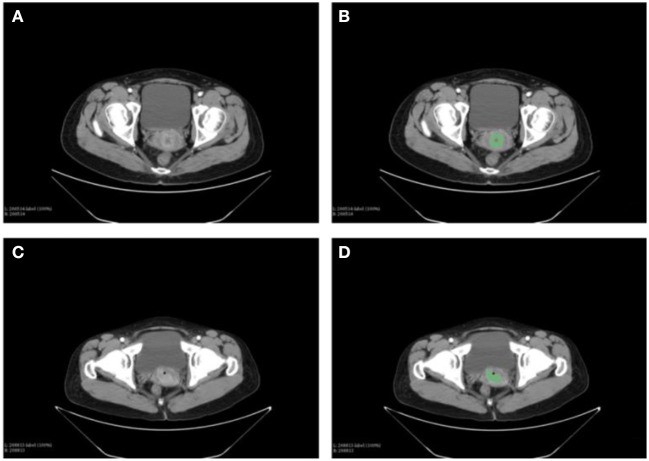
Delineation of the primary tumor on the CT images of cervical cancer patients. Two expert radiologists performed the contouring. **(A)** The CT images of primary cancer in patient 1. **(B)** The contouring of the primary cancer for patient 1. **(C)** Tumor of the patient 2 on the CT images. **(D)** Contouring of the cancer on images of patient 2.

The CT images of primary cancer for each patient were collected. The images were delineated by two expert radiologists independently (Figure 2). One hundred eighteen radiomic features had ICC more than 0.8, which would be considered as stable features and be used in the following analyses.

Then, we found that 28 features were significantly correlated with the lymph node status using he logistic regression analyses (all of them had *P* < 0.05). Five of these 28 radiomic features were selected for further study due to their higher value of C index, including wavelet-LHH_firstorder_Mean, original_glszm_GrayLevelVariance, log-sigma-3-0-mm-3D_firstorder_Skewness, original_gldm_SmallDependenceHighGrayLevelEmphasis, original_glcm_JointAverage.

**Figure 3:**
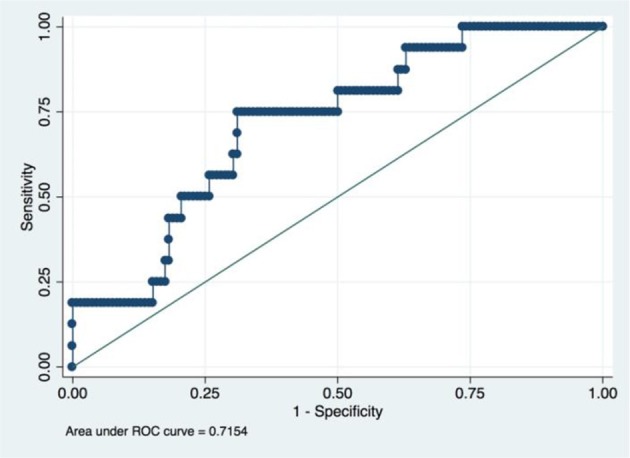
ROC of radiomic signature. Five radiomic features were selected due to their higher C index values (*P* < 0.5).

A radiomic signature was established using these five features, which had an accuracy of 88.07%, a sensitivity of 19.23%, and a specificity of 100.00%. Receiver operating characteristics (ROC) curve was also drawn for this signature, which had an area under curve (AUC) of 0.72% (*P* < 0.05) (Figure 3). Table 2 had shown the confusion matrix of this signature.

**Table 2:** Confusion matrix of the baseline and DNN model.

		**Actual class**		
		**Positive LM (% of actual)**	**Negative LM (% of actual)**	**Total**
Predicted Class	Positive LN	LRM: 7 (26.92%) SVM: 4 (15.38%) DNN: 21 (80.77%)	LRM: 0 SVM: 0 DNN: 0	
	Negative LN	LRM: 19 SVM: 22 DNN: 5	LRM: 150 (100%) SVM: 150 (100%) DNN: 150 (100%)	
	Total	26	150	176

*LM, pelvic lymph node; LRM, logistic regression model; SVM, support vector machine; DNN, deep neural network*.

### Baseline Model Based on the Conventional Machine Learning Methods

We first established a baseline model using the logistic regression model (LRM) for comparison. Recently, some researchers have found that tumor histology and grade may also have some impact on the survival of cervical cancer patients when using the larger samples from the “Surveillance, Epidemiology, and End Results Program (SEER)” database (26). Besides, deep learning models are more or less a kind of “black box” which could modulate the weights for every variable automatically upon the outcome variable. Thus, we also incorporate the tumor histology and grade into our comprehensive model.

**Figure 4:**
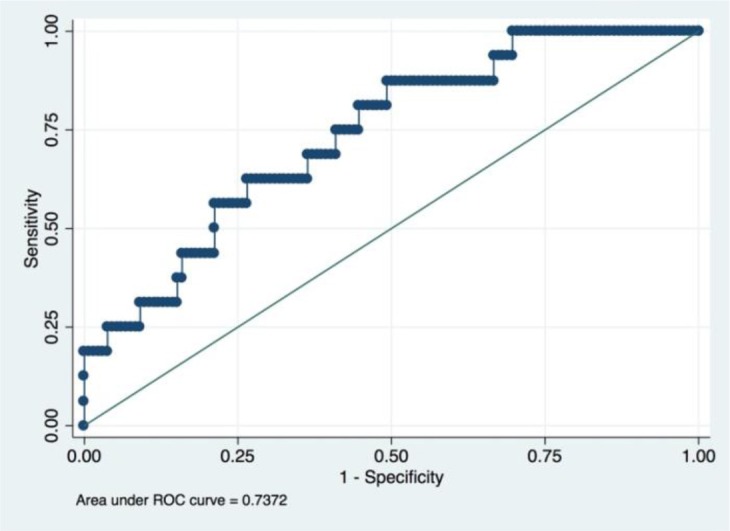
ROC of a baseline logistic regression predictive model incorporating the radiomic and clinicopathological features.

An LRM was established using he aforementioned five radiomic features, tumor histology, and grade. The final model had an accuracy of 89.20%, a sensitivity of 26.92% and a specificity of 100.00%. We had drawn the ROC curve for this model, which had an AUC of 0.74 (Figure 4). Confusion matrix of this baseline model was shown in Table 2.

**Figure 5:**
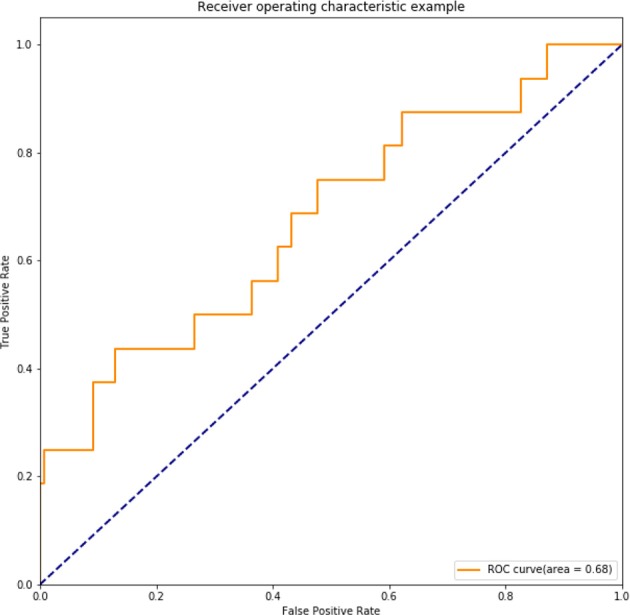
ROC of another baseline support vector machine model.

For further comparison, a support vector machine (SVM) model using these seven variables was also fitted, which had an accuracy of 87.5%, a sensitivity of 15.38%, and specificity of 100%. ROC of SVM only had 0.68 (Figure 5). The performance of the SVM was found to be worse than that of the LRM in our study, which may be due to the internal limitation of SVM for large numbers of variables. Thus, SVM would not be considered in our following study.

### A Comprehensive Model Was Established Using the Deep Learning Method

To further improve the performance of this model, DNN method was explored in this study. Aforementioned radiomic and clinicopathological features were incorporated for this comprehensive model.

Various combinations of hyperparameters had been tried for this DL model including the minibatch size, depth of the neural layers, neurons in each layer, selection of optimizer, and the number of iterations. Based on the consideration of computing cost, models that comprised more than 10,000 layers were not considered. The final DNN model was a balance between performance and computing cost.

**Figure 6:**
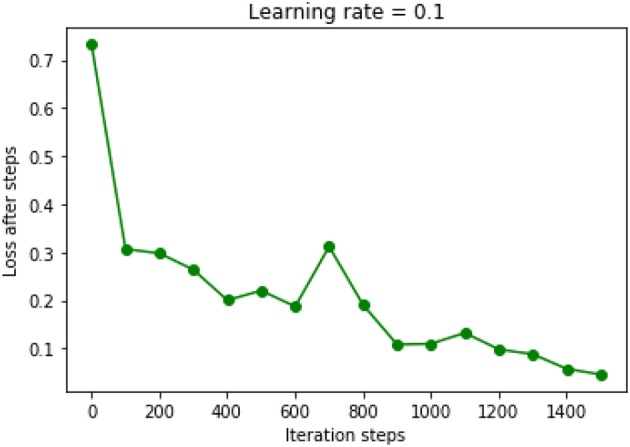
Results of cost function in the DNN model were decreased after each iteration. Learning rate was chosen as 0.1.

**Figure 7:**
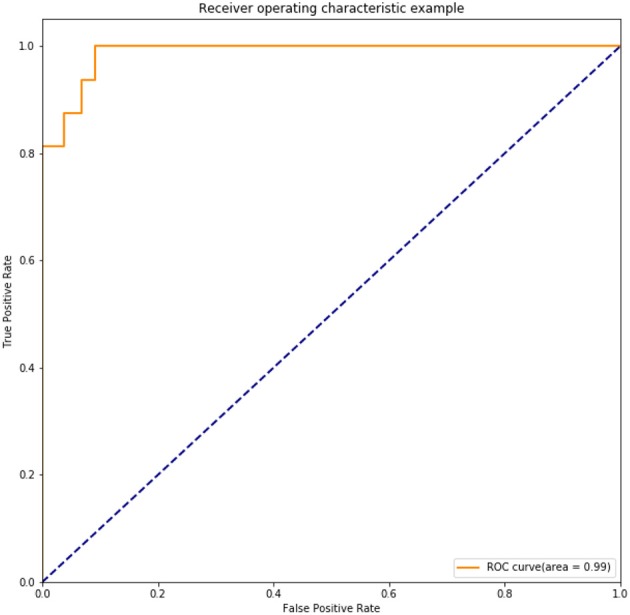
ROC of the trained DNN model after 1,500 iterations in the internal validation.

The chosen DNN model had four hidden layers, in which consisted 10 neurons. Adagrad optimizer and 1,500 iterations was used. Figure 6 showed the results of the cost function after each of the 1,500 iterations (Figure 6). This DNN had an accuracy of 97.16%, a sensitivity of 80.77%, and a specificity of 100.00%. ROC curve had a 0.99 of AUC value (Figure 7).

Further increased hidden layers or neurons in each layer could not improve the performance of this DNN model anymore. For example, when 15 hidden layers were used and each layer had 50 neurons, the accuracy would decrease to 80% of the previous performance. Besides, increased iterations could only prolong the computing time while not improving the performance of the model.

### External Validation of DNN Model Independently

**Figure 8:**
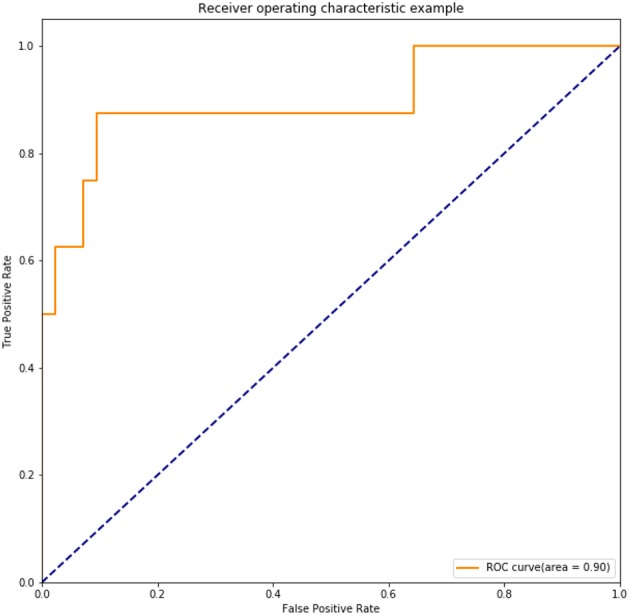
ROC of the trained DNN model tested in an independent set including 50 cases of operable cervical cancer.

Overfitting is inevitably in previous internal validation due to the limited sample size. Thus, we also performed the independent external validation in another cohort of 50 cases of operable cervical cancer patients. The clinicopathological characteristics of these 50 patients had been shown in Table 1. In this test set, DNN model still had a good precision in prediction of positive lymph node in cervical cancer patients, which achieved an accuracy of 92.00%, a sensitivity of 62.5%, a specificity of 97.62%. AUC of the ROC achieved 0.90 (Figure 8).

## Discussion

In this hypothesis generating study, we developed and validated a deep learning based radiomic model. In the independent test set, we found that this model could identify correctly in about 62.5% operable cervical cancer patients who had positive lymph nodes. To our knowledge, this is the first time that a predictive model for lymph node metastasis in cervical cancer was developed with the primary cervical cancer CT images and based on the DL method. In short, we first selected the most powerful predictive radiomic features using the LRM. We then combined these radiomic features with the clinicopathological variables to further this model's performance. These combined features were used as input data for a DNN model training. Last, the trained DNN model was proved to have 0.90 of AUC in an independent test set.

It is admitted that tumor histology and grade was not statistically significant in the univariate regression analyses in this study. A popular strategy for this situation is to rule out these variables from the final model. However, we still incorporate these two variables based on two reasons. First, statistical indifference does not mean clinical irrelevant since the so-called *p*-value are more or less influenced by the sample size of the study (27, 28). Indeed, Matsuo K et al. found that cervical cancer histology and grade could influence the survival of patients when they analyzed the really large samples of cervical patients from the SEER database (26). Our study also found that the predictive model would have better performance when these two clinicopathological factors were incorporated (AUC value increased from 0.7154 to 0.7373). Second, deep learning method is somewhat a “black box,” which means it could automatically confer the appropriate weights to corresponding variables based on the contributions made by every variable (23). Thus, upon the consideration of their easy availability, preoperative tumor histology, and grade was also incorporated in our final model.

Two different types of variables were used in this study. This multimodal combination could improve the model performance. Antoine Schernberg et al. also found that combination of neutrophil counts and SUV peak value in PET images of primary tumor could effectively predict the survival of localized advanced cervical cancer patients (14). Besides, in our previous study, we found that the incorporation of hematologic and clinical variables into the radiomic prognosis model could significantly improve the C index from 0.69 to 0.79 in NSCLC patients (15). Thus, combination of different types of biomarkers could be one of the most promising method for the further improvement of model performance in future.

Deep learning model is still a kind of “black box” in essence, and our DNN model may not be very intuitive for clinicians. However, we believe that this trend of deep learning-based prediction model is inevitable. Although other models such as the nomogram is much more visualized, nearly all of these nomograms were based on the conventional regression method which has limited power for future big data era (29). In general, human brains could only incorporate almost five variables at the same time for making reasonable decision (30, 31). Yet, with the development of cancer research, we are now accumulating more and more data from different aspects including the radiomics, genetics, clinical, pathological, etc. Thus, the use of “clinical aided diagnosis system (CADS)” is inevitable in future (32). Deep learning, broadly speaking, the artificial intelligence (AI), which has potential of great power in the clinical settings (17–19, 22, 23), would become the cornerstone of such CADS system since it could integrate different data very effectively.

There are several disadvantages in this study. First, the sample size is still small which would inevitably limit the performance of this model. Thus, we believe that performance of this model could be further improved in future if we could input more appropriate data into it. Second, we did not consider the genomic data of the biopsy of these patients. In fact, the genomic characteristics of cervical cancer tissues could be helpful for the prediction of lymph node metastases (33, 34). Thus, in our future work, we will try to incorporate more genetic information for our prediction model. Third, during the feature selection process, we only used the logistic regression method just because it is readily comprehensible. Yet, principal component analyses (PCA), LASSO regression or other machine learning methods may have equal or superior performances. We hope we could explore this issue deeper in the future studies. Forth, it is believed that performance of deep learning model could be improved with the addition of more features. Yet, in this study, we only used limited number of radiomic features just for the convenient comparison of different models. We are looking forward to establishing a more comprehensive model in future.

In summary, this study develops and validates a deep learning based radiomic model using the preoperative CT images of primary cervical cancer as well as the commonly used clinicopathological parameters. This model has been proved to have an acceptable accuracy in predicting lymph node metastases for operable cervical cancer patients.

## Data Availability Statement

The datasets generated for this study are available on request to the corresponding author.

## Ethics Statement

The studies involving human participants were reviewed and approved by The institutional review board of Shandong University. The patients/participants provided their written informed consent to participate in this study. Written informed consent was obtained from the individual(s) for the publication of any potentially identifiable images or data included in this article.

## Author Contributions

TD and XY contributed to the conception and design of this study. BC, CY, TZ, and XS contributed to data acquisition. TD and KS contributed to data interpretation and analysis. BK and XY contributed to study supervision. TD and LW contributed to manuscript editing. All authors contributed to manuscript review.

### Conflict of Interest

The authors declare that the research was conducted in the absence of any commercial or financial relationships that could be construed as a potential conflict of interest.
